# Histone H3K27 methyltransferase EZH2 regulates apoptotic and inflammatory responses in sepsis-induced AKI

**DOI:** 10.7150/thno.83353

**Published:** 2023-03-21

**Authors:** Bojun Li, Yuqi Xia, Shuqin Mei, Zehua Ye, Baofeng Song, Xinzhou Yan, Fangyou Lin, Ting Rao, Weimin Yu, Changlin Mei, Jiayi Lv, Ming Wu, Zhiguo Mao, Xiangjun Zhou, Fan Cheng

**Affiliations:** 1 Department of Urology, Renmin Hospital of Wuhan University, Wuhan, 430060, China; 2 Department of Nephrology, Shanghai Changzheng Hospital, Naval Medical University, Shanghai, 200003, China; 3 Department of Nephrology, Shuguang Hospital Affiliated to Shanghai University of Traditional Chinese Medicine, Shanghai, 201203, China

**Keywords:** EZH2, Acute kidney injury, Epigenetic, Inflammation, sepsis

## Abstract

**Rationale:** The role of histone methylation modifications in renal disease, particularly in sepsis-induced acute kidney injury (AKI), remains unclear. This study aims to investigate the potential involvement of the histone methyltransferase zeste homolog 2 (EZH2) in sepsis-induced AKI and its impact on apoptosis and inflammation.

**Methods:** We first examined the expression of EZH2 in the kidney of sepsis-induced AKI (LPS injection) mice and LPS-stimulated tubular epithelial cells. We next constructed the EZH2 knockout mice to further confirm the effects of EZH2 on apoptosis and inflammatory response in AKI. And the inflammatory level of epithelial cells can be reflected by detecting chemokines and the chemotaxis of macrophages. Subsequently, we constructed the EZH2 knocked-down cells again and performed Chromatin Immunoprecipitation sequencing to screen out the target genes regulated by EZH2 and the enrichment pathway. Then we confirmed the EZH2 target gene and its regulatory pathway* in vivo* and *in vitro* experiments. Experimental results were finally confirmed using another *in vivo* model of sepsis-induced AKI (cecal perforation ligation).

**Results:** The study found that EZH2 was upregulated in sepsis-induced AKI and that silencing EZH2 could reduce renal tubular injury by decreasing apoptosis and inflammatory response of tubular epithelial cells. EZH2 knockout mice showed significantly reduced renal inflammation and macrophage infiltration. Chromatin immunoprecipitation sequencing and polymerase chain reaction identified Sox9 as a target of EZH2. EZH2 was found to be enriched on the promoter of Sox9. Silencing EZH2 resulted in a significant increase in the transcriptional level of Sox9 and activation of the Wnt/β-catenin signaling pathway. The study further reversed the effects of EZH2 silencing by silencing Sox9 or administering the Wnt/β-catenin inhibitor icg001. It was also found that Sox9 positively regulated the expression of β-catenin and its downstream pathway-related genes. Finally, the study showed that the EZH2 inhibitor 3-deazaneplanocin A significantly alleviated sepsis-induced AKI.

**Conclusion:** Our results indicate that silencing EZH2 can protect renal function by relieving transcriptional inhibition of Sox9, activating the Wnt/β-catenin pathway, and attenuating tubular epithelial apoptosis and inflammatory response of the renal interstitium. These results highlight the potential therapeutic value of targeting EZH2 in sepsis-induced AKI.

## Introduction

Acute kidney injury (AKI) is a multifactorial-mediated kidney disease associated with a short period of kidney damage and a pronounced decrease in kidney function 1. The causes of AKI include renal ischemia, sepsis, urinary tract obstruction, and nephrotoxic drug 2. Mortality of severe AKI remains high and sepsis shows complex and unique pathophysiological mechanisms that make sepsis-induced AKI a syndrome, unlike any other AKI phenotype 3. The main pathological processes in sepsis-induced AKI are apoptosis of renal tubular epithelial cells (TECs) and inflammation in the renal interstitium. Renal insufficiency may be brought on by the renal TECs adaptive response to noxious signals, according to some studies 4, 5. Concurrent renal inflammation and microcirculatory dysfunction further amplify these processes. Novel human biopsy data confirm the function of macrophages during AKI and its development into chronic kidney disease 6.

Epigenetic mechanisms control gene expression without altering the primary nucleotide sequence, and there is increasing proof that epigenetic processes play a critical role in AKI and injury repair 7, 8. One of the main histone modifications is methylation, which can either up or down-regulate gene transcription. Lysine 27 of histone H3 (H3K27) is methylated by the protein methyltransferase enhancer of enzyme homolog 2 (EZH2), which causes the transcriptional silence of target genes 9, 10. Emerging evidence suggests that EZH2-mediated histone modifications contribute to AKI 11. Aberrant expression of EZH2 is linked to the pathogenesis of Ischemia-reperfusion and cisplatin-induced AKI 12, 13. However, there are no studies on EZH2 effects during AKI caused by sepsis, thus the potential mechanisms and the precise function of EZH2 in sepsis-induced AKI are unknown.

The Wnt/β-catenin signaling pathway is an evolutionary highly conserved signaling pathway 14, and it is involved in various physiological processes 15. Previous research has confirmed that the Wnt/β-catenin pathway has several pathogenic functions in a variety of renal disorders 16, 17, and in the current study, we demonstrated that EZH2 knockdown attenuates renal injury by upregulating Sox9 expression. Sox9 is essential for organ development, and its ongoing activation of Sox9 is the key component of renal tubular cell repair after injury 18. Regulation of the Wnt/β-catenin linked protein pathway by EZH2 was found to depend on trimethylation enrichment at position 27 of lysine in histone H3 (H3K27me3) in the promoter region, thereby reducing apoptosis and inflammation. This suggests that EZH2 may function as a cutting-edge therapeutic option in sepsis-induced AKI pathogenesis.

## Methods

### Mice

Every animal experiment complies with the demands of the Laboratory Animal Welfare and Ethics Committee of Renmin Hospital of Wuhan University. All experiments were conducted using male mice (weight 22-25 g, 6-8 weeks old, each group n=6). Wild-type (WT) mice (C57BL/6J background) were obtained from the Center of Experimental Animals of Wuhan University. A large number of studies have confirmed that EZH2 is very important for the development of mouse early embryos, and insufficient expression of EZH2 in embryos will lead to the death of mouse embryos after implantation 19, 20. Here, we conducted the tamoxifen-inducible knock out (iKO) mice, which refers to the gene knock out that depends on tamoxifen for a specific time. EZH2^fl/fl^(fl/fl) mice (Cre-negative) were provided by Pro. Xi Wang (Department of Immunology, School of Basic Medical Sciences; Advanced Innovation Center for Human Brain Protection, Beijing Key Laboratory for Cancer Invasion and Metastasis, Department of Oncology, Capital Medical University, Beijing, China.) and CAG-creER mice were generated from the mice used in our previous studies 21, 22. CAG-creEREZH2^fl/fl^ (EZH2^iKO^) mice were generated and at six weeks of age, they were injected intraperitoneally with approximately 75 mg/kg tamoxifen (T5642; Sigma-Aldrich, St. Louis, USA), once every 24 h for five consecutive days. DNA extracted from tail snips was used for PCR-based genotyping 23, 24.

### Induction of sepsis-induced AKI

We use two ways to construct mouse models. Mice were injected intraperitoneally with 15 mg/kg LPS (*Escherichia coli* 055:B5; Sigma-Aldrich) to establish an *in vivo* sepsis-induced AKI model as previously described 25. Saline solution was administered into the control mice. For cecal ligation and puncture (CLP), following anesthesia with pentobarbital 60 mg/kg. To expose the cecum, a short longitudinal midline incision was done. The cecum was subsequently ligated 1 cm from the blind-ending. The cecum was perforated by a single through-and-through puncture in a mesenteric-to-antimesenteric orientation, halfway between the ligation and the tip of the cecum. A little droplet of excrement was extruded from each penetration hole after the needle was removed. Mice that underwent the same operation but without CLP were used as a sham control. The mice were sacrificed 24 h after LPS injection or CLP, and their blood and tissues were saved for later research.

### Drug treatment

To study the effect of 3-deazaneplanocin A(3-DZNeP) (Selleckchem, Houston, USA) on sepsis-induced AKI, after injecting LPS or CLP, 3-DZNeP (2 mg/kg) was delivered intraperitoneally every day after that. Equal amounts of saline were injected into the control group. On the third day following the injection of LPS or CLP, the mice were sacrificed.

### Cell Culture

Human kidney tubular epithelial cells (HK-2 cell line) were procured from Procell Life Science & Technology Co., Ltd, Wuhan, China and THP-1 cells were procured from Stem Cell Bank, Chinese Academy of Sciences, Shanghai, China. DMEM/F12 media supplemented with 10% fetal bovine serum (Gibco, Waltham, MA, USA) and 1% antibiotics (penicillin/streptomycin) was used to cultivate HK-2 cells. Knockdown EZH2 lentivirus was purchased from Genepharma (Suzhou, China). The lentiviral sequences were: LV2-NC: TTCTCCGAACGTGTCACGT; LV2-EZH2-715: GGTGAATGCCCTTGGTCAATA; LV2-EZH2-1743: GCTCCTCTAACCATGTTTACA; LV2-EZH2-2196: GCAGCTTTCTGTTCAACTTGA. HK2 cells were infected with the virus in solution. After transfection for 72 h, cells were treated with 2 µg/mL puromycin, and stable clones were maintained at 1 μg/mL. A humidified environment with 5% CO2 was used to maintain a 37°C temperature for all cells. The siRNA was purchased from Sangon Biotech (Shanghai, China) including the siRNA targeting the human Sox9 gene (siSox9) and nontargeting siRNAs(siCon). HK-2 cells were plated in 6-well plates overnight and then transfected with siRNAs using Lipofectamine 2000(Invitrogen, USA) according to the instructions. The siRNA sequences were: siCon: sense: 5′-UUCUCCGAACGUGUCACGUTT-3′, antisense: 5′-ACGUGACACGUUCGGAGAATT-3′; siSox9: sense: 5′-GCAAGCUCUGGAGACUUCUGATT-3′, antisense: 5′-UCAGAAGUCUCCAGAGCUUGCTT-3′.

ICG-001 (Wnt/ β-catenin inhibitor, SF6827, Beyotime Biotechnology) was used at a concentration of 25 µmol/L, as described in a previous study 26.

Mouse primary renal tubular epithelial cells (mRTECs) were isolated as previously described 27.

### Macrophage Migration Assay

We used cell culture methods as published previously 28, 29. Sh-EZH2 and Sh-NC HK-2 cells were incubated with LPS (50 µg/mL), and as controls, baseline conditions were applied to sh-NC HK-2 cells. After receiving LPS treatment for 24 h, the cell supernatants were collected. In Transwell plates' top chambers, THP-1 cells were cultured (0.8 µm pore size) in 100 ng/mL phorbol-12-myristate-13-acetate (PMA) for 24 h and were then cultured in fresh complete medium without PMA for 24 h. The bottom chambers were then filled with the cell supernatants from HK-2 cells that had been treated with LPS. After co-culturing for 24 h chambers were fixed using 4% paraformaldehyde and using 0.1% crystal violet, and migrating cells were stained. Cell migration was observed by the microscope (Nikon, Tokyo, Japan).

### Cell Viability Assay and Treatment

To evaluate the effects of various LPS concentrations on cell viability, a CCK-8 assay (Dojindo Laboratories, Japan) was performed. At a density of 8,000 cells per well, HK-2 cells were seeded onto 96-well culture plates for 24 h. Cells were co-treated with various concentrations of LPS (0, 10, 20, 50, and 100 µg/mL) in DMEM for 24 h. At each time point, each well received 10 μL of the CCK-8 solution before being incubated at 37 °C for 1 h. Optical density was measured at 450 nm using a microplate reader (ELx808, BioTek Instruments, USA). Standard fitting curves were produced from the assay data, and IC50 values were calculated.

### Renal Function Measurement

Serum creatinine and urea nitrogen are used as indicators of renal function, and the corresponding kit (JianCheng, Nanjing, China) is used according to the instructions.

### Histopathological Examination

The kidney tissues were embedded in paraffin and fixed with 4% paraformaldehyde to make 2-4 μm sections. Slices of the kidney tissue underwent deparaffinization, rehydration, and hematoxylin and eosin staining. The tubular damage score was used to evaluate tissue injury as previously described 30. HE slides were scored on a scale from 0 to 4: 0, normal; 1, mild (<25% tubular damage); 2, moderate (25-49% tubular damage); 3, severe (50-75% tubular damage); 4, extensive damage (>75% tubular damage). Terminal deoxynucleotidyl transferase dUTP nick-end labeling (TUNEL) staining was used to assess degree of injury using One Step TUNEL Apoptosis Assay Kit (C1090, Beyotime), according to instructions. TUNEL-positive areas were quantified using ImageJ software (1.8.0).

### Immunohistochemistry (IHC)

Slices of a 5-mm-thick mouse kidney were dewaxed, rehydrated, and repaired in EDTA buffer before being incubated in 3% H2O2 for 10 min. Then use 5% BSA to block it for 45 min. The samples were next exposed to rabbit anti-EZH2 for overnight incubation at 4 °C. The slices were exposed to the goat anti-rabbit IgG antibody the next day for 30 min. Then counterstained sections with hematoxylin after being stained with 3,3'-diaminobenzidine. The slices were fixed, dehydrated, and dried, and then observed by microscope. Ten randomly selected fields of view from each section were used for evaluation and semi-quantitative analysis using ImageJ software (1.8.0).

### Immunofluorescence

HK-2 cells were permeabilized with 0.2% Triton X-100 after being fixed with 4% paraformaldehyde. Incubate with the following primary antibodies: EZH2 (5246S;1:100; CST), AQP-1(20333-1-AP;1:200; Proteintech), Sox9 (67439-1-Ig;1:100; Proteintech), β-catenin (GB12015; 1:100; Servicebio), F4/80 (ab6640; 1:100; Abcam), monocyte chemoattractant protein-1 (MCP-1) (GB113239; 1:100; Servicebio), surviving (10508-1-AP; 1:200; Proteintech), cyclinD1 (26939-1-AP; 1:400; Proteintech) at 4°C overnight, followed by staining with CY3- or Alexa Fluor-488-conjugated secondary antibodies (1:1000; Thermo Scientific, MA, USA). The slides were mounted for examination after being stained with 4′,6-diamidino-2-phenylindole. Using the same settings on a fluorescence microscope (Nikon).

### qPCR

Total RNA was extracted from kidney tissues with TRIzol reagent (Invitrogen Life Technologies, CA, USA) which was then reverse transcribed to create cDNA using the Takara reagent kit (Takara Biotechnology, Shiga, Japan), followed by standard methodology SYBR Green master mix (Yeasen, Shanghai, China) qPCR amplification. Relative gene expression was normalized against GAPDH. The primers used were all in Table S1.

### Western blotting analysis

Renal tissues and cells were lysed using RIPA lysate (Servicebio). The BCA method was used to quantify total protein. Proteins were separated on polyacrylamide gels , then moved to a polyvinylidene fluoride membrane 5% skim milk was used to prevent non-specific binding to the membrane for 2 h at room temperature, followed by incubation overnight with one of the following primary antibodies: EZH2 (5246S; 98 kDa; 1:1,000; CST), H3K27me3 (9733S; 17 kDa; 1:1,000; CST), H3(17168-1-AP; 17kDa; 1:2000; Proteintech), cleaved-caspase3(19677-1-AP; 17 kDa; 1:1,000; Proteintech), Bcl2 (12789-1-AP; 26 kDa; 1:1,000; Proteintech), Bax (GB114122; 21 kDa; 1:800; Servicebio), MCP-1(GB113239; 11 kDa; 266 1:500; Servicebio), Sox9 (67439-1-Ig; 56 kDa; 1:1000; Proteintech), β-catenin (GB12015; 92 kDa; 1:500; Servicebio), surviving (GB11177; 16 kDa; 1:1,000; Servicebio), GAPDH (GB12002; 37 kDa; 1:1,000; Servicebio) at 4 °C. Horseradish peroxidase-coupled secondary antibodies were incubated for 1 h with the protein bands after being washed by TBST, followed by visualization using an ECL kit (Biosharp, China) on a ChemiDoc MP system (Bio-Rad, Foster City, CA, USA). The relative density of proteins was measured using ImageJ software (1.8.0). All experiments were performed using three replicates.

### Flow Cytometry

FITC Annexin Apoptosis Detection Kit I (BD Biosciences, NJ, USA) was used to flow cytometry. Trypsin digestion of cells in 6-well plates was followed by centrifugation, resuspension in precooled PBS, and addition of 100 μL 1× binding buffer. Each tube containing cells received 5μL Annexin V-FITC for a 15 min incubation period in the dark; next, 500 μL 1× binding buffer and 4 μL propidium iodide staining solution were added, and finally, a CytoFLEX flow cytometer (Beckman Coulter, Brea, CA, USA) was used to identify the results. According to other descriptions 31, 32, kidneys were divided into pieces measuring 2~3 mm^3^ and placed in DMEM with 2 mg/mL collagenase type I for 45 min at 37 °C. After that, a BD cell strainer (40 μm) was used to filter the cell suspension. The FVS780(APC-cy7) and renal immune cell markers CD45 (FITC), CD11b (PE), and F4/80 (APC) were employed. The FlowJo program (10.8.1) was used to analyze the results of the flow cytometry.

### ELISA

IL-1, IL-6, and TNF- levels were tested using ELISA kits (Beyotime Biotechnology, China) by the manufacturer's instructions.

### Chromatin Immunoprecipitation Sequencing (ChIP-Seq) Assay

HK2 cells were crosslinked with 1% formaldehyde and sonicated to produce 200-500 bp DNA fragments for subsequent immunoprecipitation. Use 20 µg EZH2 antibodies (proteintech;21800-1-AP) and normal IgG (CST;2729s) antibody for immunoprecipitation. Libraries were produced using the KAPA HTP Library Preparation Kit (Roche, Switzerland; KK8234) according to instructions. Briefly, purified DNA fragments were end-repaired, adenylated, and ligated to adaptors, followed by PCR amplification. Libraries were subjected to high-throughput sequencing on an Illumina NovaSeq platform. Subsequent binding peak prediction analysis was performed with MACS software to identify the region of EZH2 binding 33. DeepTools was used for the assignment of genomic features, such as relative location to TSS to the peaks and visualization of binding profiles 34. HOMER (Hypergeometric Optimization of Motif EnRichment) software was used to search the enriched binding motifs in peaks 35.

### Kidney Precision Medicine Project (KPMP)

The Kidney Precision Medicine Project (KPMP) was initiated and funded by the National Institute of Diabetes, Digestive, and Kidney Diseases. KPMP uses ethical and safe kidney biopsies obtained from patients with kidney disease, combined with transcriptome, protein, metabolome, and spatial omics techniques, to produce a reference atlas of the kidney in a healthy and diseased state 36. Entering the https://atlas.kpmp.org/explorer/, enter "EZH2", select Single-Nucleus RNA-Seq (SN RNA-Seq), and select the expression data of renal tubular epithelial cells in Healthcare Reference and AKI.

### Differentially expressed genes and functional enrichment analysis

Two kinds of cells, sh-NC and sh-EZH2, were used for RNA sequencing of transcriptome: each group was repeated three times. Total RNA was extracted by TRIzol (Invitrogen, California, USA), and a cDNA library was constructed according to the manufacturer's instructions. The library was sequenced on BGISEQ500 platform (Huada Gene, China) with a clean reading depth of 10 Gb. The R Bioconductor package “edgeR” was utilized to screen out the differentially expressed genes (DEGs). The thresholds for finding DEGs were established at a false discovery rate of 0.05 and a fold change of >2 or < 0.5. KOBAS 2.0 server was used to find KEGG pathways and Gene Ontology (GO) keywords. The enrichment of each phrase was determined using the hypergeometric test and the Benjamini-Hochberg FDR controlling technique.

### Statistical analyses

Data analysis was done by GraphPad Prism software (version 9.0; GraphPad Software, CA, USA). Experiment results are shown as means standard deviation. Examining group differences involved using the Student's t test and one-way analysis of variance. P<0.05 was considered statistically significant.

## Results

### EZH2 is induced in the kidneys of mice following LPS treatment

**Figure 1:**
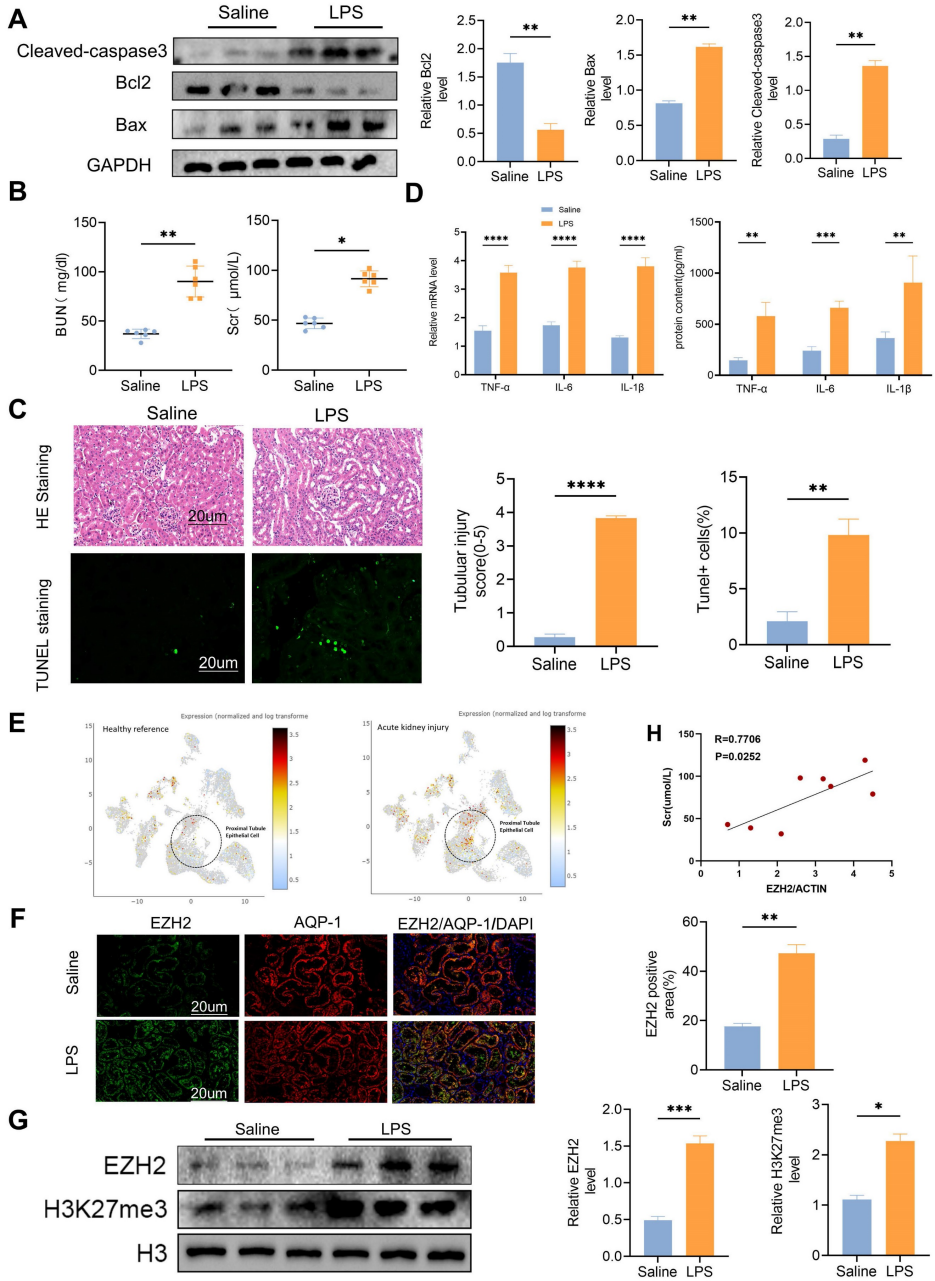
** EZH2 expression up-regulated in sepsis-induced acute kidney injury (AKI)* in vivo***. **(A)** Expression of cleaved-caspase3, Bax, Bcl2 in kidney tissue lysates from WT mice 24h after LPS (15 mg/kg) or saline injection, as detected by western blotting and quantified by densitometry. **(B)** Renal function was measured by blood urea nitrogen and serum creatinine levels of WT at 24 h after LPS (15 mg/kg) or saline injection. **(C)** Renal tubular injury was evaluated by HE staining and TUNEL in kidney tissue lysates as detected by IHC, and the percent of positive area was quantified.** (D)** The levels of TNF-α, IL-6, and IL-1β were determined by qPCR and ELISA. **(E)** Single-nucleus RNA-seq showed that EZH2 was up-regulated in the nucleus of proximal renal tubular epithelial cells of AKI. **(F)** Expression of EZH2 and proximal tubule epithelial cell marker AQP-1 in kidney tissue lysates was detected by IF, and the percent of positive area was quantified. **(G)** Expression of EZH2, H3K27me3 in kidney tissue lysates, as detected by western blotting and quantified by densitometry. **(H)** Correlation analysis of EZH2 mRNA levels and SCr levels in mice. Data represent the means ± standard error of the mean (SEM). *p < 0.05; **p < 0.01; ***p < 0.001; ****p < 0.0001.

We administered intraperitoneal injections with LPS (15 mg/kg) to the mice in the experimental group. After 24 h, western blotting showed that the expression level of apoptosis-related proteins caspase3 and Bax were activated, and anti-apoptotic protein Bcl2 was decreased (Figure 1A). The serum creatinine (SCr) and blood urea nitrogen (BUN) levels of mice in the experimental group were significantly increased (Figure 1B), and TUNEL fluorescence indicated that the *in vivo* model of sepsis-induced AKI was successfully established (Figure 1C). TNF-α, IL-6, and IL-1β were significantly up-regulated in the model group (Figure 1D). Single-nucleus RNA-seq (SNRNA-seq) showed that EZH2 was up-regulated in the nucleus of proximal renal tubular epithelial cells during AKI (Figure 1E). IF showed that EZH2 was located in proximal tubular epithelial cells and its expression was significantly up-regulated in the LPS-treated group, compared with the controls (Figure 1F). Considering the nuclear localization of EZH2 and its epigenetic mechanism, we also examined H3K27me3 expression (Figure 1G). EZH2 expression showed a positive correlation with SCr levels (Figure 1H).

### Knockdown of EZH2 attenuates sepsis-induced kidney injury in mice

**Figure 2:**
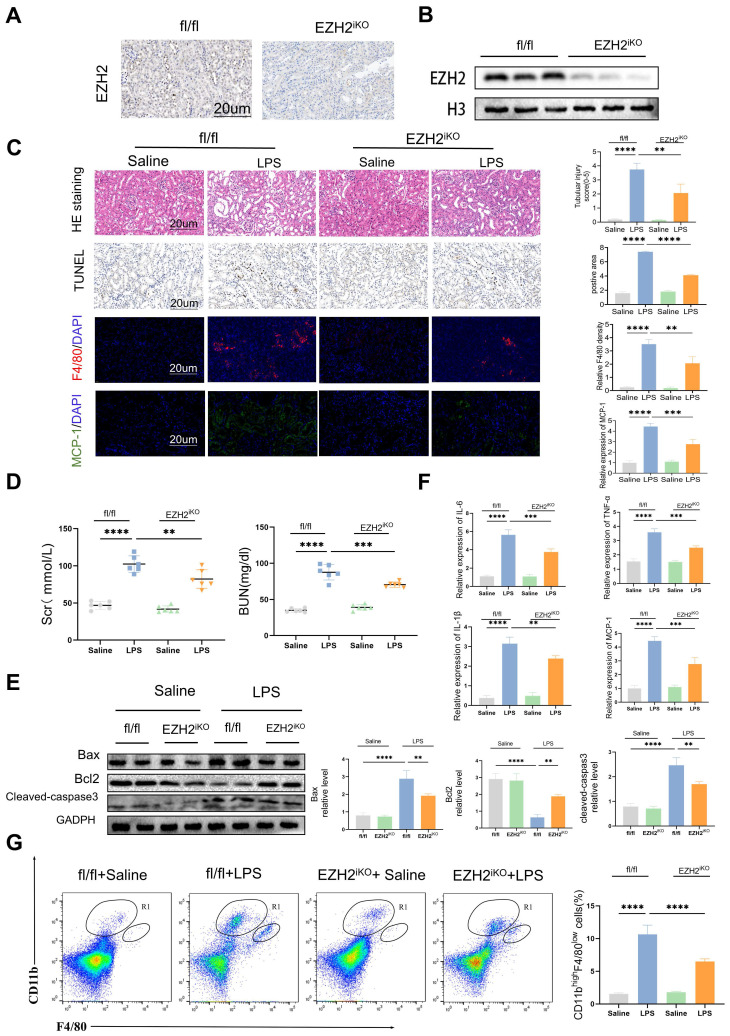
** Knockdown of EZH2 reduces tubular apoptosis and inflammation *in vivo***. **(A-B)** Expression of EZH2 in EZH2^iKO^ mice was detected by western blotting and IHC.** (C)** Renal tubular injury was evaluated by HE and TUNEL staining. The macrophage markers F4/80 and MCP-1 were detected by IF, and the percent of the positive area was quantified.** (D)** Renal function was measured by BUN and SCr levels of EZH2^fl/fl^ and EZH2^iKO^ mice at 24 h after LPS (15 mg/kg) or saline injection.** (E)** Expression of Bax, Bcl2, and cleaved-caspase3 in kidney tissue lysates from EZH2^fl/fl^ mice and EZH2^iKO^ mice at 24h after LPS (15 mg/kg) or saline injection, as detected by western blotting and quantified by densitometry. **(F)** The levels of TNF-α, IL-6, IL-1β and MCP-1 were determined by qPCR. **(G)** The proportion of macrophages in the kidney tissue of mice was detected by flow cytometry. Data represent the means ± SEMs. *p < 0.05; **p < 0.01; ***p < 0.001; ****p < 0.0001.

To further explore the biological mechanism of EZH2 in sepsis-induced AKI, we used EZH2^iKO^ mice, and knock out efficiency was tested by IHC and western blotting (Figure 2A-B). LPS caused numerous histopathological changes, including dilation of part of the renal tubular epithelium, watery degeneration, cellular or protein cast, narrowing of the lumen, and dilation of interstitial capillaries (Figure 2C). EZH2^iKO^ mice exhibited less renal damage following LPS injection, compared to EZH2^fl/fl^ mice. TUNEL histochemistry showed that apoptotic cells in the kidney tissue of EZH2^iKO^ mice were significantly smaller than those of EZH2^fl/fl^ mice. LPS caused significant increases in serum BUN and SCr levels in the AKI model, compared to the control group, and EZH2^iKO^ mice showed improved renal function and decreased expression levels of BUN and SCr (Figure 2D).

### Knockdown of EZH2 reduces apoptosis and inflammatory reaction in sepsis-induced AKI* in vivo*

Western blotting revealed that cleaved-caspase3 and Bax expression was down-regulated in EZH2^iKO^ mice, compared with that in EZH2^fl/fl^ mice, whereas Bcl2 expression was up-regulated (Figure 2E). Prior research has demonstrated that a significant mechanism of the inflammatory response in sepsis-associated AKI is the infiltration of renal interstitial immune cells, particularly the infiltration of macrophages 37. Compared with EZH2^fl/fl^ mice, EZH2^iKO^ mice exhibited significantly reduced TNF-α, IL-6, IL-1β, and MCP-1 levels (Figure 2F).

MCP-1/CCR2 is a key pathway for macrophages to respond to inflammatory responses, and the influx of macrophages is regulated by MCP-1 38, 39. IF of macrophage marker F4/80 indicated that macrophage infiltration in EZH2^iKO^ mice was significantly reduced, compared with EZH2^fl/fl^ mice, which was consistent with the trend of inflammatory factors (Figure 2C). The small population of monocytes in normal kidneys, CD11b^hi^/F4/80^lo^, significantly increases and secretes pro-inflammatory cytokines during the early inflammatory response of AKI 31, 37. Flow cytometry showed a reduced CD11b^hi^/F4/80^lo^ population in EZH2^iKO^ mice (Figure 2G). Taken together, these results suggest that the knockdown of EZH2 may play an anti-inflammatory role by reducing the accumulation of macrophages in the renal interstitium.

### EZH2 knockdown reduces LPS-induced apoptosis* in vitro*

**Figure 3:**
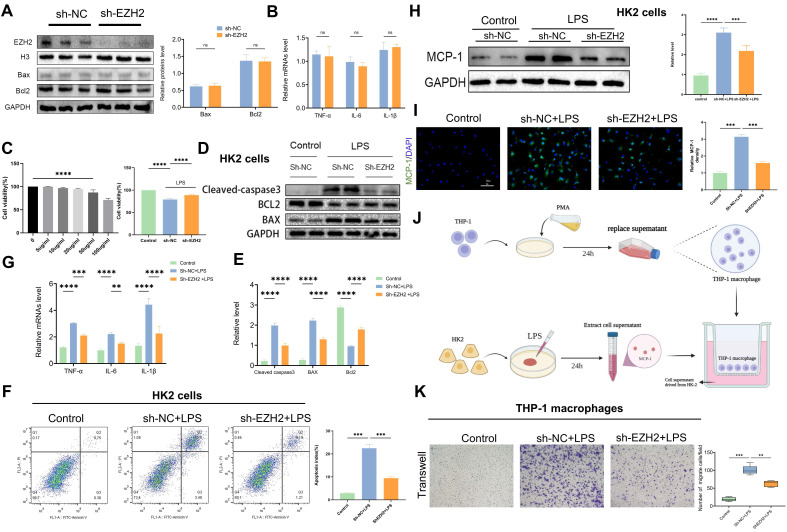
** Knockdown of EZH2 reduces apoptosis and inflammatory responses *in vitro***.** (A)** Expression of EZH2, cleaved-caspase3, Bax, and Bcl2 in HK-2 cells with or without shEZH2 was detected by western blotting and quantified by densitometry. **(B)**The levels of TNF-α, IL-6 and IL-1β were determined by qPCR.** (C)** Cell activity was determined using a CCK-8 assay under different concentrations of LPS with or without shEZH2.** (D-E)** Expression of Cleaved-caspase3, Bax, Bcl2 in HK-2 cells treated in LPS with or without shEZH2 was detected by western blotting and quantified by densitometry. **(F)** Analysis of apoptosis and apoptosis rate in HK-2 cells by flow cytometry. **(G)** The levels of TNF-α, IL-6 and IL-1β were determined by qPCR. **(H)** Expression of MCP-1 was detected by western blotting and quantified by densitometry.** (I)** The MCP-1 was detected by IF, and the percent of positive area was quantified. **(J)** Flow of THP-1 macrophage migration assay. **(K)** Transwell assays on migratory capacity of THP-1 macrophages. Data represent the means ± SEMs. *p < 0.05; **p < 0.01; ***p < 0.001; ****p < 0.0001.

To further explore the mechanism of EZH2 action in sepsis-induced AKI, we used sh-EZH2 cells. Western blotting verified knock-out efficiency of EZH2, and EZH2 shRNA has no impact on apoptosis and inflammation indexes (figure 3A-B). The CCK-8 assay revealed that at 50-100 μg/mL, cell viability was severely reduced, and the low concentration did not affect cell viability (Figure 3C). Therefore, 50 µg/mL was selected as the LPS-stimulated concentration for the *in vitro* experiment. When the EZH2 was knocked down, HK2 cell viability was restored under LPS treatment (Figure 3C). Consistent with the* in vivo* experiment, western blotting showed that the expression of cleaved-caspase3 and Bax were inhibited, and Bcl2 was up-regulated (Figure 3D-E), which agreed with the results of flow cytometry (Figure 3F). Our results were also supported by the use of mRTECs and the EZH2 inhibitor 3-DZNep (Figure S1A-B). These results suggest that inhibition of EZH2 reduces sepsis-induced AKI-induced apoptosis in epithelial cells.

### EZH2 knockdown reduces LPS-induced inflammatory response* in vitro*

According to reverse-transcription qPCR, IL-6, IL-1β, and TNF-α mRNA levels were significantly increased in HK2 cells after LPS treatment, whereas sh-EZH2 showed significantly down-regulated expression of inflammatory cytokines (Figure 3G). Infiltration of macrophages is an important feature of renal interstitial inflammatory response. Expression of MCP-1 was assessed using western blotting and IF, and in the controls, almost no MCP-1 expression was observed in HK2 cells, whereas after treatment with LPS, MCP-1 was abundantly expressed. Under EZH2 knockdown, the increase of MCP-1 was reversed (Figure 3H-I). Macrophage migration assays were performed using transwell to determine whether HK-2 cells with sh-EZH2 were effective at reducing macrophage recruitment (Figure 3J). When THP-1 cells were cultured with LPS-stimulated HK-2 cell supernatant after PMA differentiation, a significantly increased number of macrophages migrated from the upper side to the bottom. In contrast, the number of migrated macrophages was significantly reduced when exposed to the supernatant of sh-EZH2 HK-2 cells treated with LPS (Figure 3K). Results were also supported by the use of mRTECs and the EZH2 inhibitor 3-DZNep (Figure S1C). This indicates that EZH2 knock out can reduce the inflammatory response.

### EZH2 regulates the expression of Sox9 through epigenetic modification

**Figure 4:**
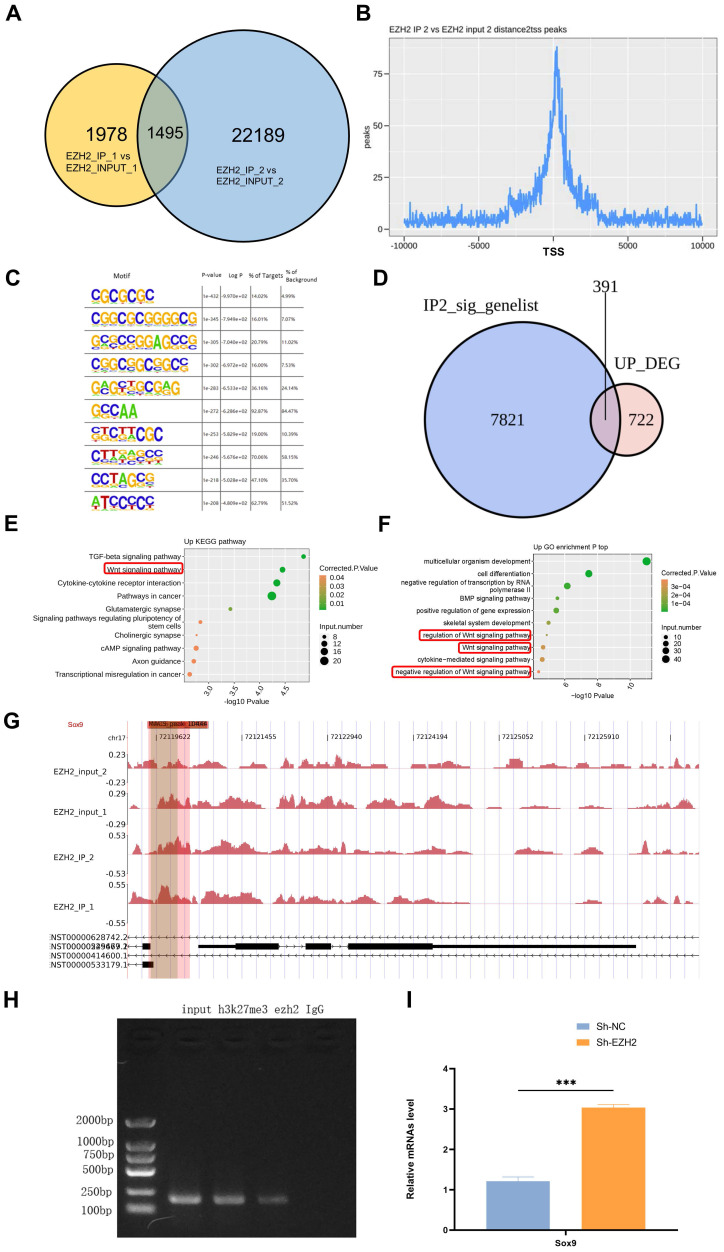
** EZH2 affects transcription of Sox9 through epigenetic effects. (A)** Overlap of EZH2 binding peaks obtained between experimental replicates. **(B)** Distribution of IP sample-specific binding peaks near the transcription start site.** (C)** Motif analysis indicates that EZH2 binds GC-rich sequences enriched. **(D)** Overlapping genes of Chip-Seq and DEG. **(E-F)** KEGG and GO enrichment analysis. **(G-H)** Enrichment of EZH2 on Sox9. **(I)** Detection of Sox9 expression level by qPCR. Data represent the means ± SEMs. *p < 0.05; **p < 0.01; ***p < 0.001; ****p < 0.0001.

**Figure 5:**
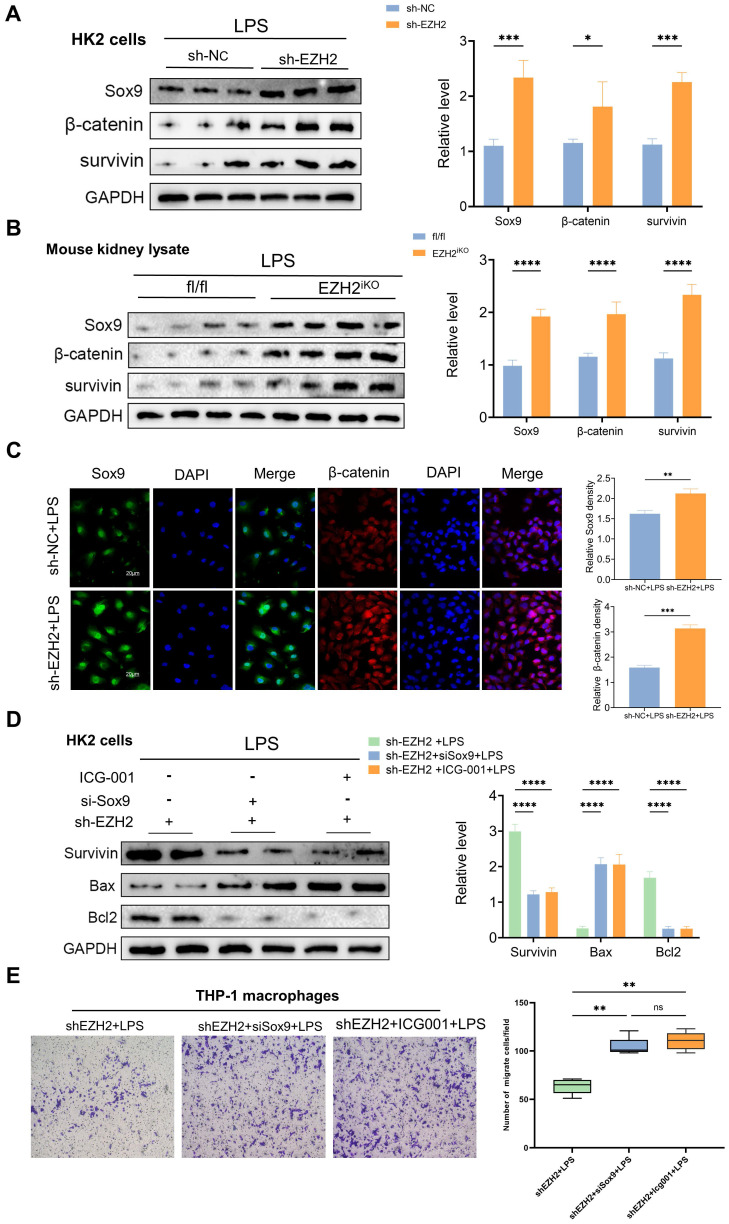
** Knockdown of EZH2 increases the expression of Sox9 and related downstream pathways. (A)** Expression of Sox9, β-catenin, and survivin in HK-2 cells treated in LPS with or without shEZH2 were detected by western blotting and quantified by densitometry.** (B)** Expression of Sox9, β-catenin, and survivin in kidney tissue lysates from EZH2^fl/fl^ mice and EZH2^iKO^ mice at 24h after LPS (15 mg/kg) or saline injection was detected by western blotting and quantified by densitometry. **(C)** Sox9 and β-catenin were detected by IF, and the percent of positive area was quantified.** (D)** Expression of β-catenin, Bax, and Bcl2 in HK-2 cells treated in LPS were detected by western blotting and quantified by densitometry. **(E)** Transwell assays on migratory capacity of THP-1 macrophages. Data represent the means ± SEMs. *p < 0.05; **p < 0.01; ***p < 0.001; ****p < 0.0001.

Two replicates of IP samples and Input samples were used, and the overlap of the EZH2 binding peaks obtained between the two experimental replicates was obtained (Figure 4A). The location distribution statistics of the binding peaks specific to IP samples were obtained according to their distance from the transcription start site, and their distribution near the transcription start site (Figure 4B). Motif analysis revealed that EZH2 is richly bound to the GC-rich sequence, suggesting that EZH2 could bind to the promoter region of the gene and thus affect the transcription of the gene (Figure 4C). We performed an overlay analysis of genes associated with EZH2 binding DNA (IP2 samples) and up-regulated genes after silencing of EZH2 in HK2 cells (Figure 4D). GO and KEGG enrichment analysis of the genes (Figure 4E-F) showed a close association with the classic Wnt signaling pathway. We sequenced 10 genes in the Wnt signaling pathway and found the binding of EZH2 to Sox9(Figure 4G). To further verify the epigenetic regulation of Sox9 by EZH2, we performed ChIP -PCR showing that EZH2 was enriched in the promoter region of Sox9 (Figure 4H). Moreover, qPCR showed that Sox9 expression was higher after EZH2 knockdown (Figure 4I). Therefore, we verified that the inhibition of EZH2 could regulate the expression of Sox9 by epigenetics to affect sepsis-induced AKI. Moreover, after EZH2 knock out, the expression of targeted molecules downstream of the Wnt/β-catenin signaling pathway was up-regulated (Figure 5A-C). Results were also supported by the use of mRTECs and the EZH2 inhibitor 3-DZNep (Figure S1D).

### EZH2 inhibition attenuates apoptosis and inflammation in sepsis-induced AKI through upregulation of Sox9

Sox9 expression was knocked down with a siRNA based on sh-EZH2 to further explore the relationship between EZH2 and Sox9, Wnt/β-catenin and apoptosis and inflammatory responses. Sox9 knockdown (siSox9) led to down-regulated expression of survivin, downstream genes of the Wnt/β-catenin pathway. The downregulation of Bax was reversed, as was the down-regulation of anti-apoptotic protein Bcl2. The expression of inflammatory factors was significantly increased, such as that of MCP-1, and the migration ability of macrophages was reversed. Similarly, we used ICG-001 (a Wnt/β-catenin pathway inhibitor), and the apoptosis-related proteins, and macrophage migration were also reversed (Figure 5D-E). When EZH2 was knocked down, Sox9 expression was restored, and the expression of Wnt/β-catenin pathway was activated, which helps alleviate kidney damage. Transwell assays also suggested a reversal of macrophage migration following the same knockdown of Sox9 and administration of Wnt/β-catenin pathway inhibitors. EZH2 directly acted on Sox9 to regulate the Wnt/β-catenin pathway and thus affected the apoptosis and inflammatory response during sepsis-induced AKI.

### Sox9 regulates apoptosis and inflammatory response via the Wnt/β-catenin pathway

**Figure 6:**
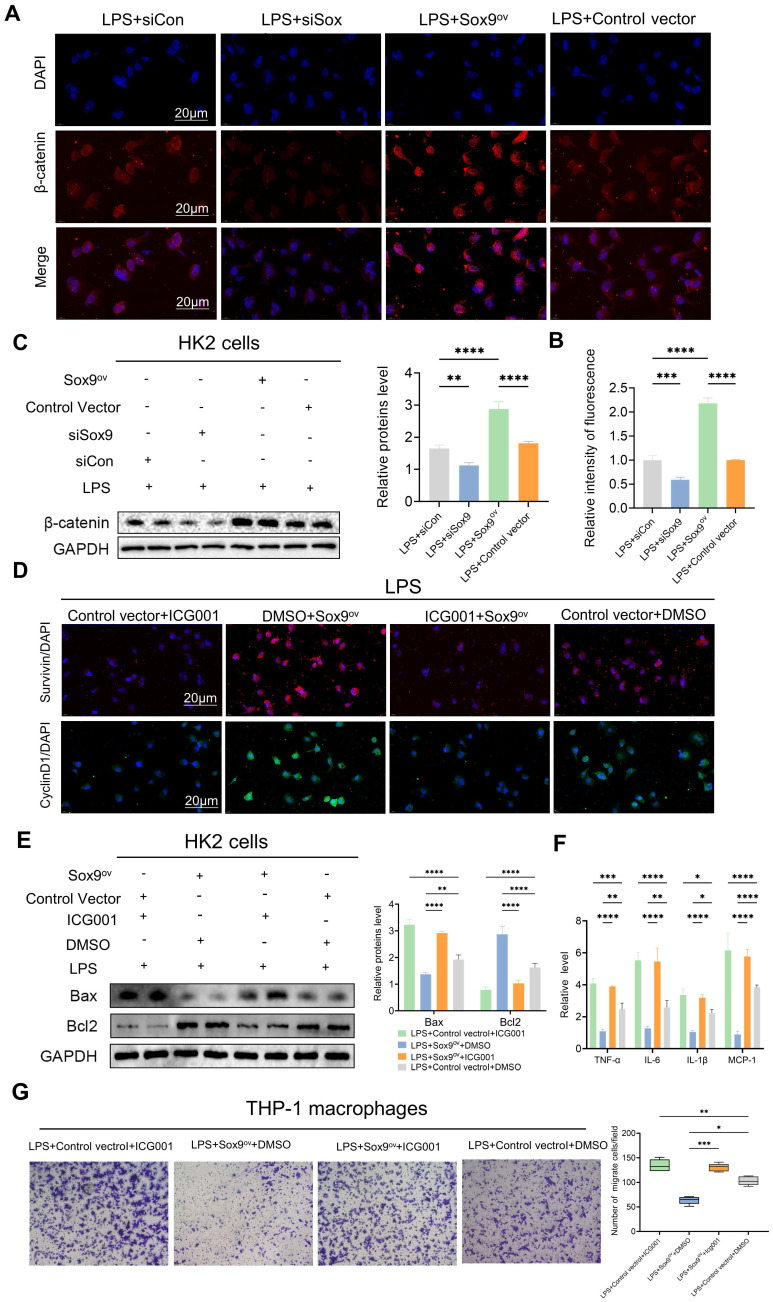
** Sox9 regulates apoptosis and inflammatory response via the Wnt/β-catenin pathway. (A-B)** Detection of β-catenin in HK-2 cells by IF and the percent of positive area was quantified with overexpression (Sox9^ov^) and knockdown of Sox9(si-Sox9). **(C)** Expression of β-catenin was detected by western blotting and quantified by densitometry. **(D)** Survivin and CyclinD1, the downstream genes of β-catenin, were detected by IF. **(E)** Expression of Bax and Bcl2 was detected by western blotting and quantified by densitometry. **(F)** The levels of TNF-α, IL-6, IL-1β and MCP-1 were determined by qPCR. **(G)** Transwell assays on migratory capacity of THP-1 macrophages. Data represent the means ± SEMs. *p < 0.05; **p < 0.01; ***p < 0.001; ****p < 0.0001.

To further investigate the relationship between Sox9 and the Wnt/β-catenin pathway, we explored changes in β-catenin and downstream phenotype by overexpression (Sox9^ov^) and knockdown of Sox9. We found that when Sox9 was overexpressed, the expression of β-catenin increased and nuclear translocation occurs. When Sox9 was knockdown, the expression of β-catenin decreased (Figure 6A-C). Overexpression of Sox9 also increased the expression of survivin and CyclinD1, downstream genes of the Wnt/β-catenin pathway, which were inhibited by ICG001(Figure 6D). While Sox9 overexpression reduced the expression of apoptotic and inflammatory factors and inhibited the recruitment of macrophages, the Wnt/β-catenin pathway inhibitor ICG001 offset the effects of Sox9 overexpression (Figure 6E-G).

### EZH2 inhibitor 3-deazaneplanocin A (3-DZNep) protects renal functioning in sepsis-induced AKI

**Figure 7:**
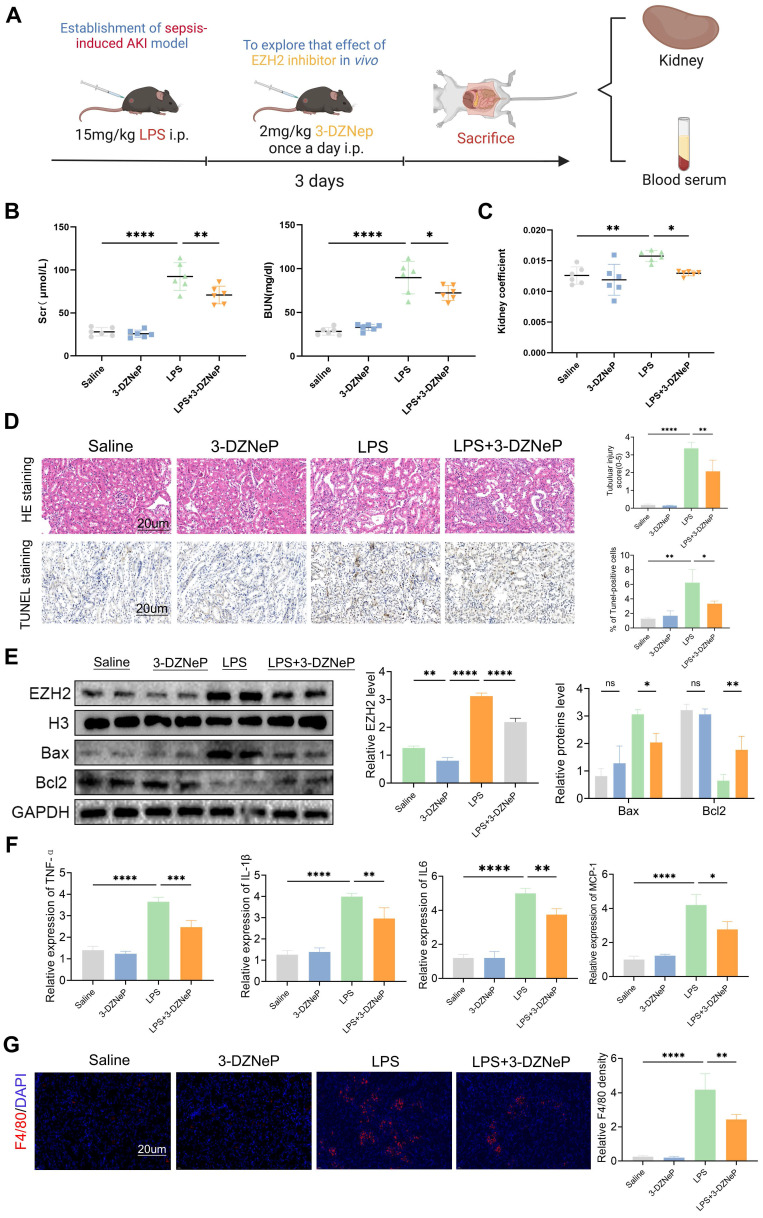
** EZH2 inhibitor 3-Deazaneplanocin A (3-DZNep) protects the renal function of in sepsis-induced AKI by inhibiting apoptosis and inflammation. (A)** Experimental flow chart. **(B-C)** BUN and SCr levels and kidney coefficient in mice. **(D)** HE staining and TUNEL staining of the kidneys. **(E)** Expression of EZH2, Bax, and Bcl2 in kidney tissue lysates from mice. **(F)** The levels of TNF-α, IL-6, IL-1β, and MCP-1 were determined by qPCR. **(G)** Detection of macrophage marker F4/80 in mouse kidney tissue by IF. Data represent the means ± SEMs. *p < 0.05; **p < 0.01; ***p < 0.001; ****p < 0.0001.

Inhibitor of EZH2, 3-DZNep, was used to validate the function of EZH2 *in vivo* during AKI (Figure 7A), and SCr and BUN levels and kidney coefficients were decreased in the LPS+3-DZNep group, and pathological renal damage was alleviated (Figure 7B-D). In addition, TUNEL staining showed that 3-DZNep reduced apoptosis in renal cells. This was consistent with Western blotting, where 3-DZNep inhibited EZH2 expression and reduced Bax expression restoring Bcl2 expression (Figure 7E). 3-DZNep consistently down-regulated levels of MCP-1, IL-6, IL-1β, and TNF-α (Figure 7F). IF showed that the EZH2 inhibitor reduces the infiltration of macrophages (Figure 7G). To further validate our experimental results, CLP was used to construct the sepsis AKI model (Figure S2A-C). EZH2 was significantly up-regulated in the CLP model, and 3-DZNep inhibited the expression of EZH2. HE and TUNEL staining showed that 3-DZNep reduced apoptosis in renal cells. IF showed that the EZH2 inhibitor reduces the infiltration of macrophages (Figure S2D). Western blotting also supports the above results (Figure S2E). 3-DZNep consistently down-regulated levels of MCP-1, IL-6, IL-1β, and TNF-α (Figure S2F-G). These data generally imply that 3-DZNep protects against AKI and reduces inflammatory reaction, indicating that EZH2 is a critical factor in AKI.

## Discussion

EZH2 is generally modified by epigenetic modifications to inhibit the transcription of a target gene and this mechanism has been studied predominantly in tumors 40. In the current study, we focused on the mechanism of EZH2 in sepsis-induced AKI. Numerous previous studies have shown that EZH2 is involved in the regulation of inflammation 41, which may be related to the pathophysiology of sepsis. Particularly, during sepsis-induced acute lung injury, after inhibiting EZH2, inflammation and lung injury are significantly alleviated 42. The kidney-protective effects of EZH2 inhibition have been demonstrated in several investigations recently, inhibition of EZH2 was shown to reduce oxalate-induced kidney injury by modulating the JNK/FoxO3a pathway 43, and inhibition of EZH2 prevents cisplatin-induced renal tubular apoptosis and AKI by restoring E-calmodulin expression 44. Moreover, inhibition of EZH2 can attenuate I/R-induced AKI by reducing reactive oxygen species 45. However, the direct mechanisms of EZH2 during AKI are unclear.

Dysfunctional inflammatory responses are involved in the pathophysiology of septic AKI 46, and circulating inflammatory cytokines increased the risk of death in patients with AKI 47. Specifically, by interacting with a number of transmembrane receptors, these pro-inflammatory cytokines might enhance the inflammatory response brought on by early mediators 46. In addition, excessive inflammatory infiltration can directly affect the renal parenchyma and promote the apoptosis of renal tubular cells, thereby inducing the development of AKI. It is noteworthy that renal TECs are critical mediators of macrophage recruitment and the ensuing renal inflammatory cascade 48. The pathophysiology and development of sepsis-induced AKI are greatly aided by inflammation defined by macrophage infiltration. Numerous studies have confirmed that macrophages are recruited from the circulatory system to the injured site to participate in inflammatory reactions during AKI, which is strongly related to the prognosis of AKI, and the migration ability of macrophages is an important evaluation index 6, 29, 42.This results confirmed that EZH2 deficiency reduced apoptosis of renal TECs and inhibited the tubulointerstitial inflammation characterized by macrophage infiltration.

Using pathway enrichment analysis on the genes selected by ChIP-seq and DEGs, we found that the Wnt pathway might be involved in the physiological process of EZH2 regulating AKI. In the two IPs, some top common genes were identified in descending order according to the abundance of peaks, and Sox9 was predominant, which was consistent with the results reported above. Previous studies have confirmed the protective effects of Sox9 in AKI, and the most important mechanism is involved in the process of post-acute kidney injury repair 18. Growth factors mediate many cellular responses after AKI, including repair and injury responses. Many of these growth factors, such as Wnt/β- catenin, are essential for the development of kidneys but are barely active in intact adult kidneys and are re-expressed after injury. This study found knocking down EZH2 reduces the level of H3K27me3 on Sox9 and thus promotes the expression of this gene. Under the stimulation of LPS, the existence of EZH2 enriched on Sox9 inhibits its transcription, while after the knock-down of EZH2, the inhibition of Sox9 is eliminated. The epigenetic regulation of EZH2 on Sox9 has been demonstrated, and inhibition of EZH2 has been previously reported to improve the degradation of cartilage endplates by relieving transcriptional inhibition of Sox9 49.

The action mechanism of Wnt/β-catenin pathway is very complex. When the Wnt signaling pathway is activated, the Wnt protein combines with the transmembrane receptor Frizzled/LRP 5/6 on the cell membrane to activate the Dsh protein in the cytoplasm, which inhibits phosphorylation and degradation of β-catenin by the cleavage complex, resulting in increased concentrations of β-catenin in the cytoplasm and its entry into the nucleus 15. The mechanism of the Wnt/β-catenin signaling pathway during AKI remains controversial, and it is considered a double-edged sword in AKI 50. However, increasing evidence suggests that activation of the Wnt/β-catenin pathway has a kidney-protective effect in the early pathological process of AKI, and β-catenin plays a key role in reducing the apoptosis of renal tubular epithelial cells in the acute phase of AKI, and study have demonstrated that Sox9 can continuously activate the wnt/β-catenin pathway to protect the IR-AKI 26, 51-53.In addition, the Wnt/β-catenin pathway is closely related to the inflammatory response 53. Sox9 was released after transcriptional inhibition of EZH2, and western blotting, as expected, showed up-regulation of the expressions of β-catenin and survivin downstream of the Wnt/β-catenin signaling pathway. To further confirm the direct action mechanism of Sox9, we performed transient knockdown of Sox9 in sh-EZH2 cells, and western blotting revealed that the inhibition of apoptosis-related proteins was reversed, and the migration ability of macrophages representing the interstitial inflammatory response was also reversed. Moreover, expression of Wnt/β-catenin signaling pathway was reduced, which was consistent with the result given to icg001. These results consistently demonstrated that knockdown of EZH2 activated the Wnt/β-catenin signaling pathway by up-regulating the expression of Sox9, and it inhibited apoptosis and inflammatory responses. However, according to previous studies, the long-term activation of this pathway may be related to the progression of AKI to chronic kidney disease, and whether EZH2 is involved in this process needs further investigation 50.

The development of AKI may be associated with a complex cascade of physiopathological mechanisms and may not be determined by one specific process. Therefore, we investigated the damage to TECs and the expression of related inflammatory factors and chemokines, as well as the migratory capacity of macrophages. Whether there is direct "communication" between epithelial cells and macrophages remains to be investigated, including whether macrophages affect the damage of epithelial cells, and some studies suggest that epithelial cells secrete exosomes that affect the polarization level of macrophages 54, which are directions for future research.

Our results clearly correlate cell death-immune crosstalk with the development of AKI and demonstrate that knock-down of EZH2 get involved in sepsis-induced AKI by up-regulating the transcription of Sox9. Targeting the EZH2/Sox9 signaling pathway may provide new strategies for preventing or ameliorating sepsis-induced AKI.

## Supplementary Material

Supplementary figures and table.
