# Supplementary material for: Histone H3K27 methyltransferase EZH2 regulates apoptotic and inflammatory responses in sepsis-induced AKI
Source: Theranostics. 2023 Mar 21;13(6):1860–75. doi: 10.7150/thno.83353 (PMC10091874; doi:10.7150/thno.83353)
Supplement: Supplementary file 1 — Supplementary figures and table. [file thnov13p1860s1.pdf]

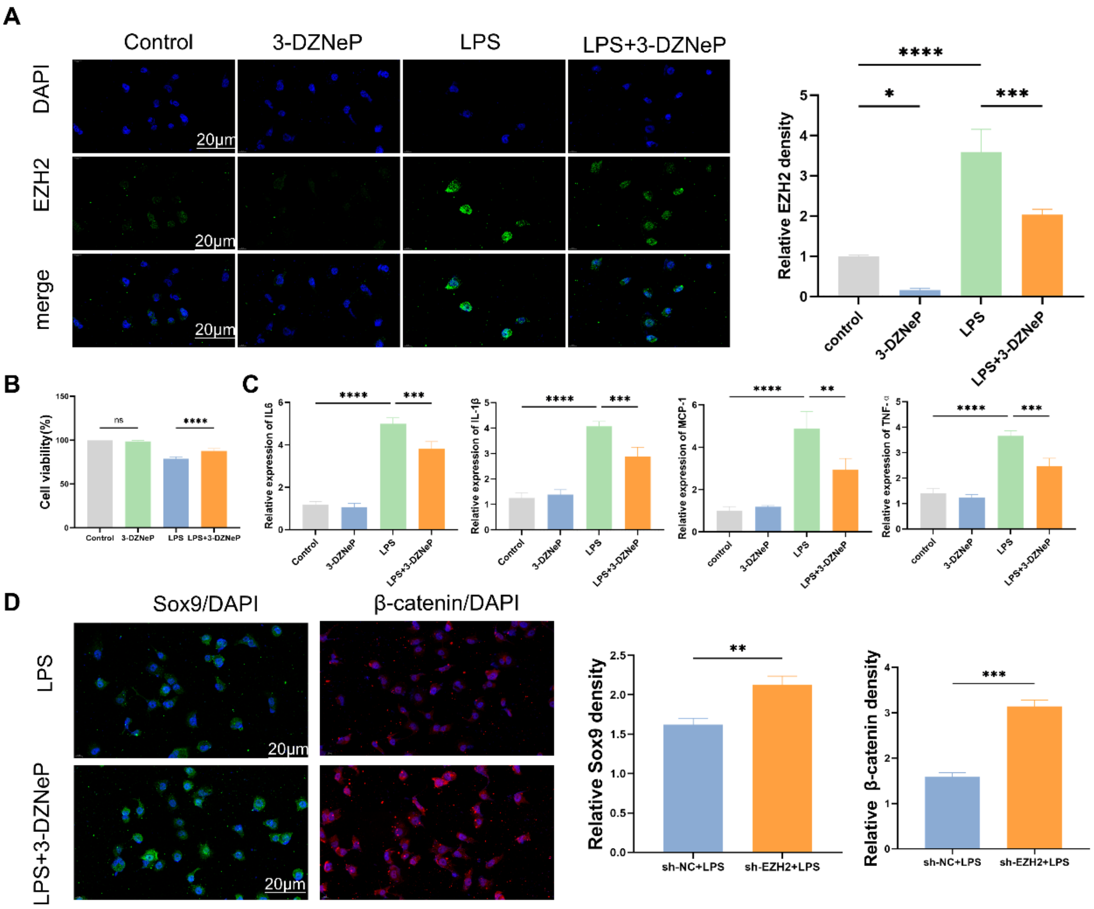

Figure S1. EZH2 inhibition reduces injury by regulating Sox9/β-catenin in primary murine renal tubular epithelial cells (mRTECs). (A) Expression of EZH2 in mRTECs with or without 3-DZNep was detected by IF. **(B)** Cell activity was determined using a CCK-8 assay under LPS with or without 3-DZNep. **(C)** The levels of MCP-1, TNF-α, IL-6, and IL-1β were determined by qPCR. **(D)** Expression of Sox9 and β-catenin in mRTECs with or without 3-DZNep was detected by IF. Data represent the means ± SEMs. *p < 0.05; **p < 0.01; ***p < 0.001; ****p < 0.0001.

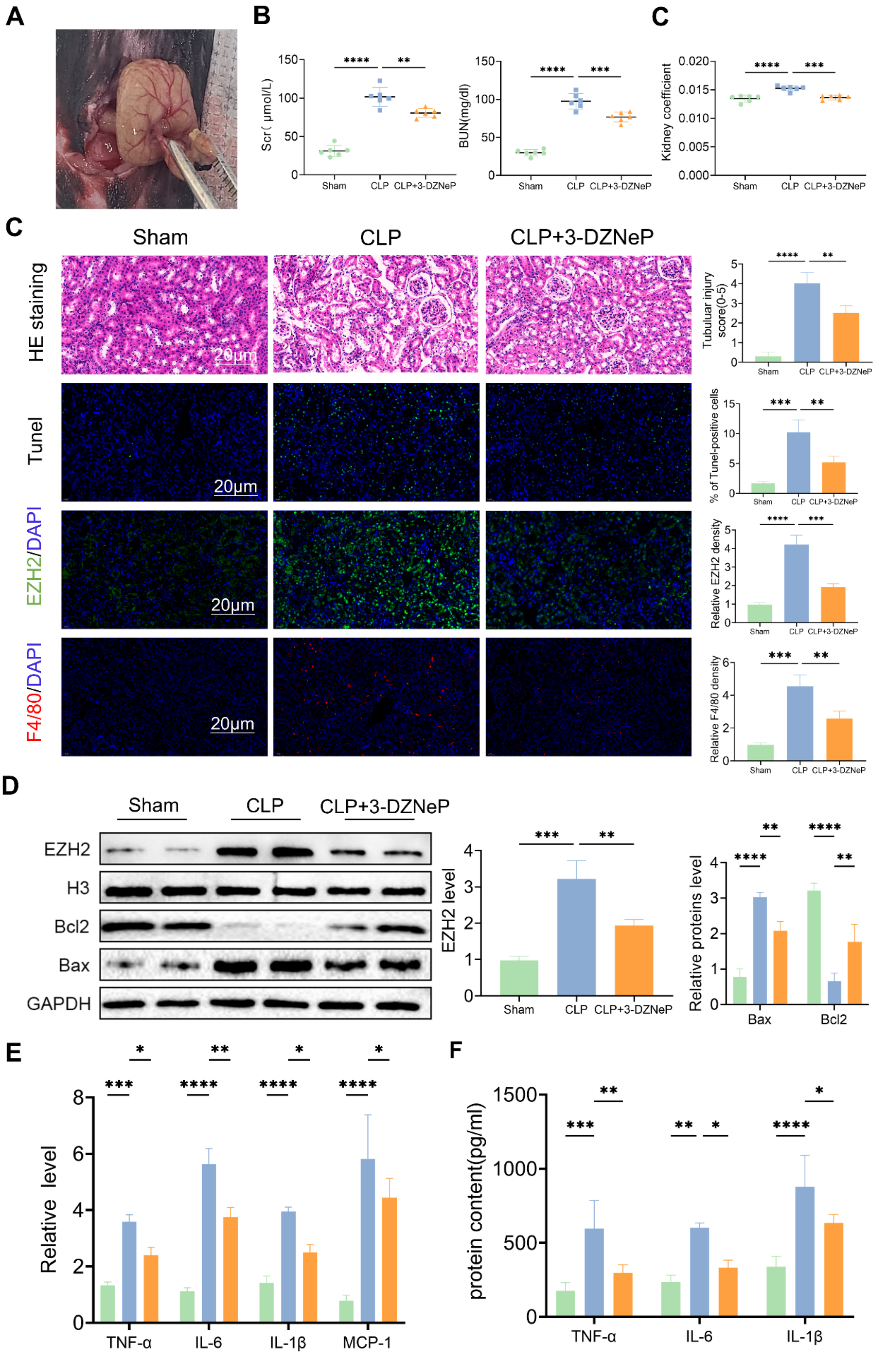

Figure S2. EZH2 inhibitor 3-DZNep protects the renal function of AKI in CLP model. (A) Establishment of cecal ligation and puncture model. **(B-C)** BUN and SCr levels and kidney coefficient in mice. **(D)** HE staining, TUNEL staining of the kidneys and EZH2, F4/80 in mouse kidney tissue were

detected by IF. **(E)** Expression of EZH2, Bax, and Bcl2 in kidney tissue lysates from mice. **(F-G)** The levels of TNF-α, IL-6, IL-1β and MCP-1 were determined by qPCR and ELISA. Data represent the means ± SEMs. *$p < 0.05$; **$p < 0.01$; ***$p < 0.001$; ****$p < 0.0001$.

Table S1 List of primers used for qRT-PCR

Target		Sequence 5’→3’
M-IL-1β	Forward	GGGCCTCAAAGGAAAGAATCT
	Reverse	GAGGTGCTGATGTACCAGTTGG
M-IL-6	Forward	CTGGGAAATCGTGGAAATGAG
	Reverse	AAGGACTCTGGCTTTGTCTTTCT
M-TNF-α	Forward	TCCCCAAAGGGATGAGAAGTT
	Reverse	GAGGAGGTTGACTTTCTCCTGG
M-MCP-1	Forward	TAAAAACCTGGATCGGAACCAAA
	Reverse	GCATTAGCTTCAGATTTACGGGT
M-EZH2	Forward	AGCACAAGTCATCCCGTTAAAG
	Reverse	AATTCTGTTGTAAGGGCGACC
M-GAPDH	Forward	TGCTGTCCCTGTATGCCTCTG
	Reverse	TTGATGTCACGCACGATTTCC
H-IL-1β	Forward	CGATCACTGAACTGCACGCTC
	Reverse	ACAAAGGACATGGAGAACACCACTT
H-IL-6	Forward	CAATGAGGAGACTTGCCTGGTG
	Reverse	TGGCATTTGTGGTTGGGTCA
H-TNF-α	Forward	TCTACTCCCAGGTCCTCTTCAAG
	Reverse	GGAAGACCCCTCCCAGATAGA
H-MCP-1	Forward	AGCAGCAAGTGTCCCAAAGA
	Reverse	TTGGGTTTGCTTGTCCAGGT
H-EZH2	Forward	AATCAGAGTACATGCGACTGAGA
	Reverse	GCTGTATCCTTCGCTGTTTCC
H-Sox9	Forward	AGCGAACGCACATCAAGAC
	Reverse	CTGTAGGCGATCTGTTGGGG
H-GAPDH	Forward	GGAAGCTTGTCATCAATGGAAATC
	Reverse	TGATGACCCTTTTGGCTCCC
